# Arabidopsis leucine-rich repeat receptor–like kinase NILR1 is required for induction of innate immunity to parasitic nematodes

**DOI:** 10.1371/journal.ppat.1006284

**Published:** 2017-04-13

**Authors:** Badou Mendy, Mary Wanjiku Wang’ombe, Zoran S. Radakovic, Julia Holbein, Muhammad Ilyas, Divykriti Chopra, Nick Holton, Cyril Zipfel, Florian M. W. Grundler, Shahid Siddique

**Affiliations:** 1 Rheinische Friedrich-Wilhelms-University of Bonn, INRES – Molecular Phytomedicine, Bonn, Germany; 2 The Sainsbury Laboratory, Norwich Research Park, Norwich, United Kingdom; The Ohio State University, UNITED STATES

## Abstract

Plant-parasitic nematodes are destructive pests causing losses of billions of dollars annually. An effective plant defence against pathogens relies on the recognition of pathogen-associated molecular patterns (PAMPs) by surface-localised receptors leading to the activation of PAMP-triggered immunity (PTI). Extensive studies have been conducted to characterise the role of PTI in various models of plant-pathogen interactions. However, far less is known about the role of PTI in roots in general and in plant-nematode interactions in particular. Here we show that nematode-derived proteinaceous elicitor/s is/are capable of inducing PTI in Arabidopsis in a manner dependent on the common immune co-receptor BAK1. Consistent with the role played by BAK1, we identified a leucine-rich repeat receptor-like kinase, termed NILR1 that is specifically regulated upon infection by nematodes. We show that NILR1 is essential for PTI responses initiated by nematodes and *nilr1* loss-of-function mutants are hypersusceptible to a broad category of nematodes. To our knowledge, NILR1 is the first example of an immune receptor that is involved in induction of basal immunity (PTI) in plants or in animals in response to nematodes. Manipulation of NILR1 will provide new options for nematode control in crop plants in future.

## Author summary

Host perception of pathogens via receptors leads to the activation of antimicrobial defence responses in all multicellular organisms, including plants. Plant-parasitic nematodes cause significant yield losses in agriculture; therefore resistance is an important trait in crop breeding. However, not much is known about the perception of nematodes in plants. Here we identified an Arabidopsis leucine-rich repeat receptor-like kinase, NILR1 that is specifically activated upon nematode infection. We show that NILR1 is required for the induction of immune responses initiated by nematodes and *nilr1* loss-of-function mutants are hypersusceptible to a broad category of nematodes. Manipulation of NILR1 will provide new options for nematode control in crop plants in the future.

## Introduction

Plant-parasitic nematodes attack the majority of economically significant crops, as shown by international surveys indicating an overall yield loss of 12%. In some crops, such as banana, a loss of up to 30% has been reported. Losses amount to $100 billion annually worldwide [1]. The economically most important nematodes belong to the group of sedentary endoparasitic nematodes that includes root-knot nematodes (*Meloidogyne spp*.) and cyst nematodes (*Globodera spp*. and *Heterodera spp*.). Most chemical pesticides used for control of plant-parasitic nematodes are environmentally unfriendly, expensive and ineffective in the long term. Therefore, an increased demand for novel crop cultivars with durable nematode resistance is inevitable [2, 3]. In this context, it is important to identify and characterize the different natural means by which plants defend themselves against nematodes.

The infection cycle for root-knot and cyst nematodes begins when second-stage juveniles (J2) hatch from eggs. J2, the only infective stage, search for roots guided by root exudates. They invade the roots by piercing the epidermal root cells using a hollow spear-like stylet. After entering the roots, they migrate through different cell layers until they reach the vascular cylinder. There, root-knot nematodes induce the formation of several coenocytic giant cells, whereas cyst nematodes induce the formation of a syncytium. Because established juveniles become immobile, the hypermetabolic and hypertrophic feeding sites serve as their sole source of nutrients for the rest of their lives. In a compatible plant-nematode interaction, plant defence responses are either down-regulated or overcome by the nematodes [4–6]. A cocktail of secreted molecules including effectors that are synthesized in the oesophageal glands of the nematodes is purportedly responsible for modulating the plant defences as well as the induction and development of the syncytium [7–10]. Whereas most root-knot nematodes reproduce parthenogenically, cyst nematodes reproduce sexually. Although the mechanism of sex determination in cyst nematodes is not clear, studies have shown that the majority of juveniles develop into females under favourable nutritional conditions. When juveniles are exposed to adverse growth conditions, as it is the case with resistant plants, the number of male nematodes increases considerably [11].

Numerous studies have shown that plants sense microbes through the perception of pathogen/microbe-associated molecular patterns (PAMPs or MAMPs) via surface-localised pattern recognition receptors (PRRs), leading to the activation of PAMP-triggered immunity (PTI). The activation of PTI is accompanied by the induction of an array of downstream immune responses including bursts of calcium and reactive oxygen species (ROS), cell-wall reinforcement, activation of mitogen-associated and calcium-dependent protein kinases (MAPKs and CDPKs), and massive reprogramming of the host transcriptome [12–15]. Together, these downstream responses can fend off the pathogen’s infection. PAMPs are typically evolutionary conserved across a class of pathogens and perform an important function in the pathogen life cycle [16].

Plant PRRs are either plasma membrane-localised receptor-like kinases (RLKs) or receptor-like proteins (RLPs) [14]. Both RLKs and RLPs consist of an extracellular receptor domain (ECD) for ligand perception, a single membrane-spanning domain, but only RLKS have a cytoplasmic kinase domain. The major classes of RLKs are leucine-rich repeat (LRR)-RLKs, lysine-motif (LysM)-RLKs, crinkly4 (CR4)-RLKs, wall-associated kinases (WAKs), pathogenesis-related protein 5 (PR5)-RLKs and lectin-RLKs (LeCRKs). Nevertheless, it is becoming increasingly clear that PRRs do not act alone but are part of multiprotein complexes at the plasma membrane [13]. For example, the LRR-RLK BRASSINOSTEROID INSENSITIVE-1 (BRI1)-ASSOCIATED KINASE 1 (BAK 1) forms receptor complexes with various LRR-containing PRRs to positively regulate PTI [14–15, 17]. In addition to PAMPs, plant PRRs can also perceive endogenous molecules, so-called damage-associated molecular patterns (DAMPs) that are released upon cell damage or pathogenic attack [16].

Although extensive studies have been conducted to characterise the role of PTI response in various models of plant-pathogen interactions, relatively less information is available pertaining to nematode-induced PTI responses in plants. To date, no PRR that recognises a nematode-associated molecular pattern (NAMP) has been identified [18]. However, some recent work suggests that nematode infection triggers PTI responses in host through surface-localised receptors. For example, silencing of the orthologues of BAK1 in tomato (*Solanum lycopersicum*, *Sl*) (*SlSERK3A* or *SlSERK3B)* has been shown to increase the susceptibility of these plants to nematodes due to defects in activation of basal defence [19]. In a more recent publication, it was shown that nematode infection triggers PTI responses in Arabidopsis in a BAK1-dependent and BAK1-independent manners. These authors showed that several PTI-compromised mutants including *bak1-5* were significantly more susceptible to root-knot nematodes as compared to control [20]. However, the identity of ligands and/or receptors involved in BAK1-mediated response remains unknown. As far as NAMP identification is concerned, ascarosides, which are conserved nematode-secreted molecules, have been shown to elicit plant defence responses that lead to reduced susceptibility against various pathogens [21].

In comparison to PTI, Effector-triggered immunity (ETI) during plant-nematode interaction is relatively well studied. A number of host resistance genes (*R-genes*) against nematodes have been described and their mode of action is relatively well investigated [22]. Notably, a host cell-surface immune receptor Cf-2 has been shown to provide dual resistance against a parasitic nematode *Globodera rostochiensis* and a fungus *Cladosporium fulvum* through sensing perturbations of the host-derived protease RCR3 by the venom allergen-like protein of *Globodera rostochiensis* [23]. In the present study, we provide evidence that nematodes induce PTI-like responses in Arabidopsis that rely on the perception of elicitors by membrane-localised LRR-RLKs.

## Results

### Nematode infection triggers PTI responses in host plants

**Fig 1:**
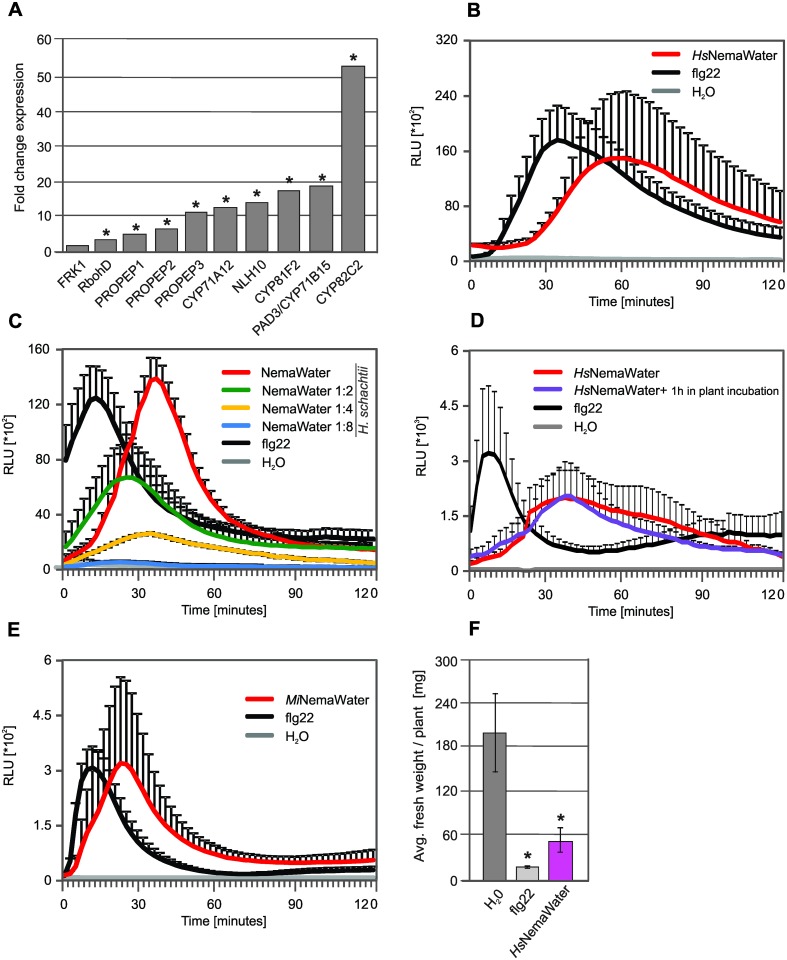
Nematode infection induced defense responses in plants that are characteristics of PTI. **(A)** Expression of PTI marker genes in microarray analysis upon nematode infection in migratory stage. Root segments from uninfected roots were used as control. Values indicate fold change compared with control. Asterisk indicates significant difference to control *(FDR* <0.05; Fold change >1.5). (**B**) Root segments from Col-0 plants were treated with water, *Hs*NemaWater or flg22 and ROS burst was measured using L-012 based assay from 0 to 120 min. (**C**) Root segments from Col-0 plants were treated with water, different dilutions of *Hs*NemaWater or flg22 and ROS burst was measured using L-012 based assay from 0 to 120 min. (**D**) Root segments from Col-0 plants were incubated with *Hs*NemaWater for 1 hour and then this *Hs*NemaWater was used for production of ROS burst on fresh root segments. Water, fresh *Hs*NemaWater or flg22, were used as controls. (**E**) Root segments from Col-0 plants were treated with water, *Mi*NemaWater, or flg22 and ROS burst was measured using L-012 based assay from 0 to 120 min. (**B-E**) Bars represent mean ± SE for three technical replicates. Experiment was repeated three times with same results. RLU, relative light units. (**F**) 5-day-old Col-0 seedlings were incubated in water, *Hs*NemaWater or flg22 for seven days. Fresh weight was measured at 12 days after germination. Data were analysed using *t-test*. Asterisk represent significant difference to water-treated control root segments (P<0.05). Hs, *Heterodera schachtii*. Mi, *Meloidogyne incognita*.

To reveal changes in gene expression in response to nematodes at and around the infected area, GeneChip analysis was performed. Small root segments (approx. 0.5 cm) containing nematodes that were still in their migratory stage (defined as continuous stylet movement), were cut and compared with corresponding root segments from plants that were not infected. Total RNA was extracted, labelled, and amplified to hybridize with the GeneChip Arabidopsis ATH1 Genome (Affymetrix UK Ltd). The ATH1 Genome Array contains more than 22,500 probe sets representing approximately 24,000 genes. Subsequent analysis of the data showed that approximately 2,110 genes were differentially expressed (*FDR* < 0.05; Fold change > 1.5). Among them, 1,139 were upregulated, whereas 971 were downregulated (S1 Data). To explore regulation of the biological processes, molecular functions, and their distribution across different cellular components, a gene ontology enrichment analysis was performed on significantly upregulated genes. Those categories which were particularly over-represented in the differentially upregulated genes included the immune system response, response to stimulus, death, and the regulation of the biological processes (Fig A in S1 Text). We have previously published a subset of 62 genes representing selected jasmonic acid (JA), ethylene (ET) and salicylic acid marker (SA), signalling and biosynthesis genes from this GeneChip data, which were also validated by qRT-PCR [24]. In general, transcript levels of genes involved in JA/ET signalling and biosynthesis were increased. However, in comparison to JA/ET, changes in SA-related genes were relatively less pronounced. Nevertheless, a slight increase in a SA biosynthesis (PAL1) and few SA signalling genes (NPR1, NPR3) was also observed (S2 Data). A detailed look at the transcriptomic data indicate that nematode infection triggered the induction of genes previously shown to be induced during PTI (Fig 1A) [25–27].

### NemaWater elicits PTI responses in host plants

Our transcriptome data showed the induction of PTI-like responses upon nematode infection, however, it was unclear whether this induction was due to the recognition of nematodes by plant receptors or whether it was the result of wounding due to continuous nematode movement. To clarify this, we established a PTI screening assay involving the measurement of ROS burst, one of the hallmark responses of PTI. For this purpose, we incubated the pre-infective J2 of *H*. *schachtii* in H_2_O for 24 hours at RT. The water obtained after removing the nematodes was termed as NemaWater (*Heterodera schachtii* NemaWater, *Hs*NemaWater; *Meloidogyne incognita* NemaWater, *Mi*NemaWater) and was used to treat Arabidopsis roots (see Methods for details). After treatment, ROS burst was measured using a root-based procedure adapted from a previous work [27]. Flg22 and H_2_O treatments were used as positive and negative controls, respectively. Treatment with flg22 as well as with *Hs*NemaWater induced a strong and consistent ROS burst in roots (Fig 1B). The ROS burst with *Hs*NemaWater was, however, slightly delayed as compared to flg22; the ROS burst to flg22 occurs within 10 to 40 min, while that to *Hs*NemaWater occurred after 20 to 120 min. Although *Hs*NemaWater induced a consistent ROS burst in Arabidopsis roots, it was not clear whether this is due to the presence of a NAMP in *Hs*NemaWater or whether it is due to the production of an eliciting-molecule by plants (upon NemaWater treatment), which in turn induced production of ROS burst in roots. Such an eliciting-molecule could be called as DAMP or a NIMP (nematode-induced molecular pattern). One way to address the question of NAMP, or DAMP/NIMP was to dilute the *Hs*NemaWater with H_2_O and analysed the production of ROS burst in roots. We hypothesised that if ROS burst is due to production of a DAMP or NIMP, diluting the NemaWater would not only reduce the magnitude of the ROS burst but may also slow its kinetics. However, our data showed that although magnitude of ROS burst was reduced strongly upon dilution, there was no delay in production of ROS between different dilutions (Fig 1C). Next, we incubated the *Hs*NemaWater with Arabidopsis roots for 60 min and then used this *Hs*NemaWater for production of ROS burst on fresh roots. The data showed that prior incubation of *Hs*NemaWater with roots did not cause any significant change in magnitude as well as kinetics of ROS Burst (Fig 1D). Regardless of the nature or origin of elicitor, activation of ROS burst upon *Hs*NemaWater treatment confirmed our observations from transcriptomic studies indicating that PTI-like responses are induced upon nematode detection.

**Fig 2:**
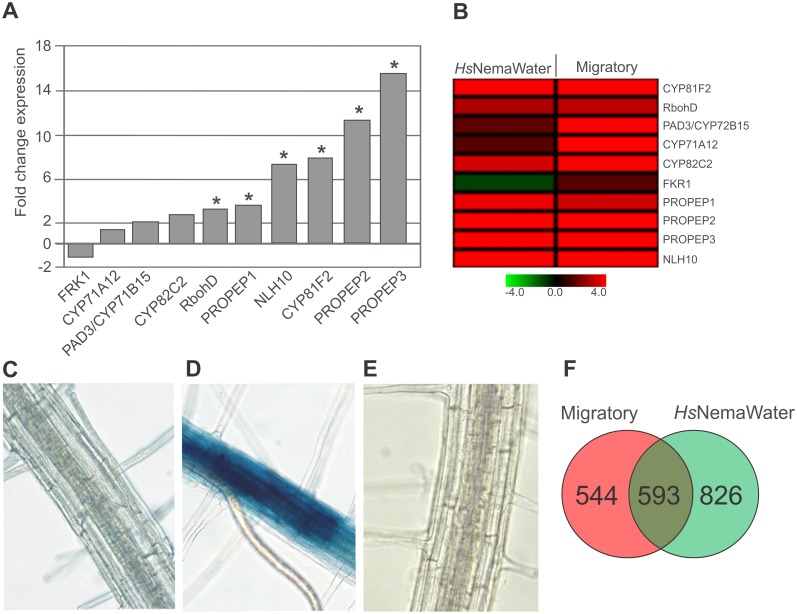
NemaWater treatment induced defense responses in plants that are characteristics of PTI. **(A)** Expression of PTI marker genes in microarray analysis upon *Hs*NemaWater treatment. Root segments from uninfected roots were used as control. Asterisk indicates significant difference to control *(FDR* <0.05; Fold change >1.5). (**B**) A heatmap showing expression of PTI marker genes upon nematode infection or upon *Hs*NemaWater treatment. (**A-B**) Values represent fold change compared with control. (**C-E**) Expression of glucuronidase (GUS) driven by *pCYP71A12* in control (C), *H*. *schachtii* infection at migratory stage (D) and *Hs*NemaWater treated plants (E) (**F**) A Venn diagram showing distribution of upregulated genes in Arabidopsis upon nematode infection or upon *Hs*NemaWater treatment.

To confirm whether NemaWater from different species of nematodes elicit a similar response, we produced NemaWater from the root-knot nematode species, *Meloidogyne incognita* (*Mi*NemaWater) and performed ROS burst assays. We observed a strong and consistent ROS burst (Fig 1E) similar to that of *H*. *schachtii* (Fig 1B). A prolonged treatment of young Arabidopsis seedlings with flg22 activated defense responses and leads to growth inhibition [28]. Although the mechanism underlying this growth inhibition is unclear, it is commonly accepted that activation of defense responses may take the resources away from growth. Importantly, this assay has frequently been used to analyse the eliciting capacity of PTI components [28, 29]. We tested whether NemaWater also caused seedling growth inhibition, and found that both flg22 and *Hs*NemaWater inhibited seedling growth and reduced the root weight to a similar extent (Fig 1F, Fig B in S1 Text). Our results suggest that NemaWater contains potential elicitor/s that is/are recognized by an immune receptor in plants leading to the activation of PTI-like responses. To test this hypothesis, we incubated 12-day-old Arabidopsis seedlings in *Hs*NemaWater for one hour: ddH_2_O alone was used as a control. RNA was extracted from the roots of both the non-treated control and NemaWater-treated seedlings. They were subsequently labelled, amplified, and hybridized with a GeneChip, as described above. The data analysis showed that 2,520 genes were differentially expressed, of which, 1,422 were upregulated and 1,098 were downregulated (*FDR* < 0.05; Fold change > 1.5; S3 Data). A gene ontology enrichment analysis for differentially upregulated genes showed the over-representation of categories such as immune system response, response to stimulus, death, signaling and the regulation of the biological processes (Fig C in S1 Text). A look at the expression of hormonal response gene upon *Hs*NemaWater treatment showed the same tendency for upregulation of JA/ET-related genes as observed upon nematode infection as described above (S2 Data). Moreover, a significant increase in the expression of genes characteristics for PTI was detected (Fig 2A). This upregulation in expression of PTI marker genes was very similar to that observed upon infection with nematodes (Fig 2B). Interestingly, expression of camalexin biosynthesis genes (*PAD3/CYP71B15*, *CYP71A12*) was upregulated only in nematode-infected plants but was not regulated upon *Hs*NemaWater treatment (Fig 2B). This was further confirmed by analyzing a reporter line (*pCYP71A12*:GUS) [30] on treatment either with nematodes or with *Hs*NemaWater. We found a strong GUS expression upon nematode infection, whereas such an expression was absent in seedlings treated with *Hs*NemaWater (Fig 2C–2E). We validated the microarray data by measuring the expression of 13 genes via qRT-PCR upon treatment with *Hs*NemaWater. Our analysis showed a similar trend for expression of selected genes as shown by microarray data (Table 1). Together, these results suggest that both nematode infection and NemaWater treatment induce PTI responses including a significant activation of JA pathways. The data analysis also showed that the changes in gene expression triggered upon treatment of seedlings with *Hs*NemaWater were to an extent similar to those that were observed upon nematode infection (Fig 2F and S4 Data). Even so, both treatments induced expression of a distinct set of genes, which may reflect differences in both treatments such as number and concentration of elicitors, duration of treatments, physical damage, etc.

**Table 1:** Validation of changes in gene expression upon *Hs*NemaWater treatment via qRT-PCR. The values represent relative fold change in response to NemaWater treatment as compared with control roots. 18S was used as housekeeping gene to normalize the data. All values are means of three biological replicates +/- SD.

Locus	GeneChip	qRT-PCR	Function
	Fold Change Control vs *Hs*NemaWater treated roots		
At3g55950	2.2	3.6 +/- 1.6	Crinkly4 Related 3
At4g21390	8.3	6.9 +/- 2.51	B120: serine/threonine kinase
At1g66880	4.3	5.3 +/- 1.1	Protein kinase superfamily protein
At1g69930	38.4	38.1 +/- 6.2	Glutathione-s-transferase 11
At3g46230	36.4	34.2 +/- 18.7	Heat shock protein 17.4
At2g38470	12.6	10.0 +/- 7.7	WRKY33
At5g25930	6.0	5.22 +/- 0.3	LRR-RLK, Protein phosphorylation
At4g23190	5.2	5.38 +/- 1.1	Cysteine-rich-RLK
At1g74360	4.1	3.28 +/- 2.2	Nematode-Induced-LRR-RLK 1
At5g48540	3.7	3.03 +/- 1.3	RLK-family protein
At1g11050	3.6	2.52 +/- 0.9	ATP-binding protein kinase
At1g61590	-2.4	-1.56 +/- 0.28	Defense response protein kinase
At4g26790	-2.5	-9.3 +/- 6.6	GDSL-motif esterase/lipase

**Fig 3:**
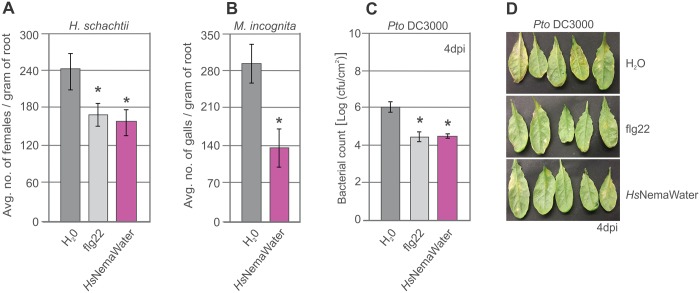
Pre-treatment with NemaWater induces resistance to pathogens. (A-B) Roots of Col-0 plants were treated with water or *Hs*NemaWater prior to infection and number of females were counted at 14 dai for cyst nematodes and number of galls were counted at 19 dai for root-knot nematodes. Bars represent mean ± SE for three independent biological replicates. (C-D) Plants were sprayed with flg22 or *Hs*NemaWater prior to inoculation and C. F.U/cm^2^ was counted at 4 dai. Bars represent mean ± SE. Experiments were repeated three times with similar results. Asterisks represent significant difference to water-treated control root segments (P<0.05).

On the basis of our finding that NemaWater triggers PTI responses, we asked whether pre-treatment with NemaWater effects plant responses to nematodes and other pathogens. To test this, plants were pre-treated with *Hs*NemaWater 24 hours prior to inoculation and were then infected with juveniles of *H*. *schachtii or M*. *incognita* or the virulent bacterial pathogen *Pseudomonas syringae* pv. tomato (see Methods for details). We found a strong decrease in number of nematodes in *Hs*NemaWater-treated plants compared with Col-0 (Fig 3A and 3B, Fig D in S1 Text). Similarly, the growth of virulent *P*. *syringae* was also reduced strongly upon *Hs*NemaWater treatment (Fig 3C and 3D).

### NemaWater-induced PTI responses are mediated by BAK1

**Fig 4:**
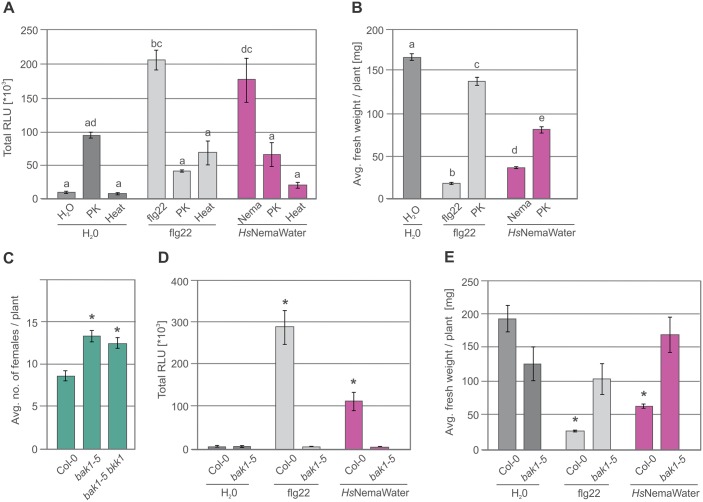
NemaWater treatment induced PTI responses were reduced strongly upon proteinase K, heat treatment, and in *bak1-5* plants. (**A**) Effect of Proteinase K and heat on production of ROS burst in root segments from Col-0 plants treated with water, *Hs*NemaWater or flg22. ROS burst was measured by using L-012 based assay from 0 to 120 min. PK, Proteinase K. Bars represent mean ± SE for two independent biological replicates. Data were analysed using single-factor ANOVA and Tukey’s post hoc test (P<0.05). Columns sharing same letter are not statistically different. (**B**) 5-day-old Col-0 seedlings were incubated in water, *Hs*NemaWater, or flg22 with or without Proteinase K for seven days. Fresh weight was measured at 12 days after germination. Bars represent mean ± SE for two independent biological replicates. Data were analysed using single-factor ANOVA and Tukey’s post hoc test (P<0.05). Columns sharing same letter are not statistically different. (**C**) Average number of female nematodes per plant in Col-0, *bak1-5* and *bak1-5 bkk1*. (**D**) Root segments from Col-0 and *bak1-5* plants were treated with water, *Hs*NemaWater or flg22 and ROS burst was measured using L-012 based assay from 0 to 120 min. (**E**) 5-days-old Col-0 and *bak1-5* seedlings were incubated in water, *Hs*NemaWater or flg22 for seven days. Fresh weight was measured at 12 days after germination. (**C-E**) Bars represent mean ± SE for three independent biological replicates. Data were analyzed using single-factor ANOVA and Dunnet post hoc test. Asterisks represent significant difference to control (P<0.05).

Induction of PTI by NemaWater indicated the presence of putative elicitor(s) in NemaWater. To test whether these elicitors is/are of proteinaceous nature, we added Proteinase K to *Hs*NemaWater and performed a ROS production assay. Duration and intensity of NemaWater-induced ROS burst varied in different experimental batches, which may be due to differences in the concentration of elicitors in different preparations of NemaWater and the possibility that NemaWater may contain more than one elicitor. Therefore, we used total photon count as a more reliable parameter for quantification of ROS burst activation in this study. We observed that the treatment of *Hs*NemaWater with Proteinase K or heat strongly reduced the induction of ROS burst (Fig 4A). These results were further confirmed by seedling growth inhibition assays (Fig 4B). BAK1 has been shown to act as a co-receptor for LRR-RLKs and LRR-RLPs, which typically detect proteinaceous ligands [14, 15]. Considering the data from Proteinase K treatment (Fig 4A and 4B) and recently published data on root-knot nematodes [20], we hypothesized that *bak1* mutants would be more susceptible to cyst nematodes. A nematode infection assay was performed on *bak1-5* and the double mutant *bak1-5 bkk1-1* (BKK1 being the closest homolog of BAK1) [31]. Both mutants were significantly more susceptible to nematodes compared with Col-0, as they allowed more females to develop (Fig 4C). We also investigated whether BAK1 is required for PTI-responses upon *Hs*NemaWater treatment and found that the nematode-derived ROS burst was strongly reduced in *bak1-5* mutants (Fig 4D). Similar results were obtained in seedling growth inhibition assays (Fig 4E and Fig E in S1 Text).

### Nematode-triggered PTI is mediated by LRR-RLK NILR1

**Fig 5:**
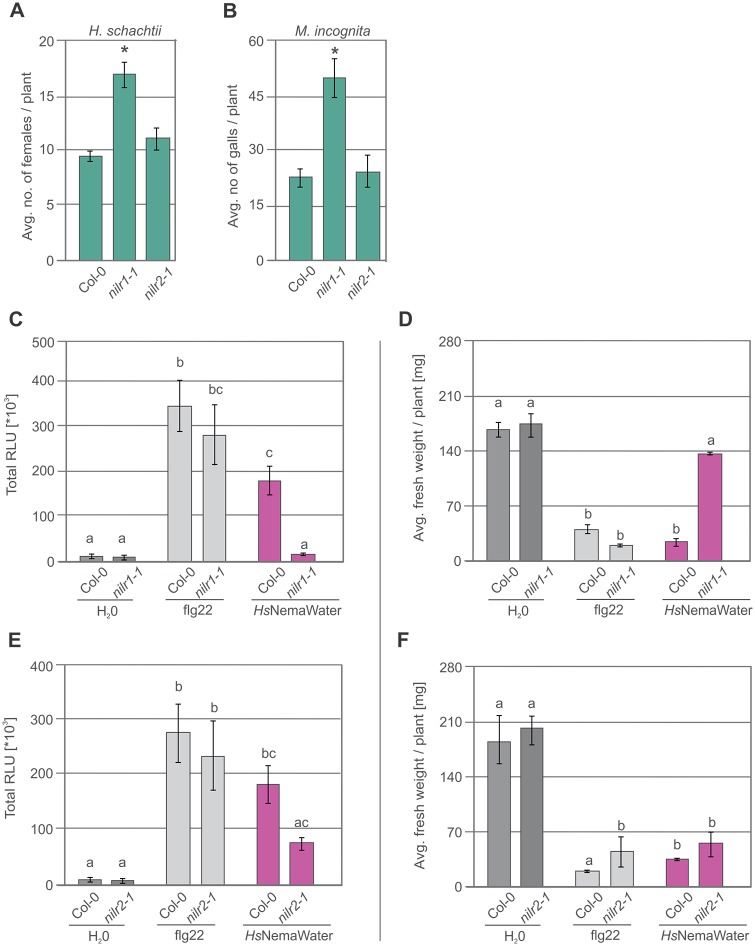
Knock-out *nilr1-1* enhances susceptibility to nematodes. (**A**) Average number of female nematodes induced by *H*. *schachtii* per plant in Col-0, *nilr1-1* and *nilr2-1*. Bars represent mean ± SE for three biological replicates. (**B**) Average number of galls induced *by M*. *incognita* per plants in Col-0, *nilr1-1* and *nilr2-1*. Bars represent mean ± SE for three biological replicates. (**C**) Root segments from Col-0, and *nilr1-1* plants were treated with water, *Hs*NemaWater or flg22 and ROS burst was measured using L-012 based assay from 0 to 120 min. Bars represent mean ± SE for sixteen biological replicates. (**D**) 5-day-old Col-0 and *nilr1-1* seedlings were incubated in water, *Hs*NemaWater, or flg22 for seven days. Fresh weight was measured at 12 days after germination. Bars represent mean ± SE for three independent biological replicates. (**E**) 5-day-old Col-0 and *nilr2-1* seedlings were incubated in water, *Hs*NemaWater, or flg22 for seven days. Fresh weight was measured at 12 days after germination. Bars represent mean ± SE for three independent biological replicates. (**F)** Root segments from Col-0 and *nilr2-1* plants were treated with water, *Hs*NemaWater or flg22 and ROS burst was measured using L-012 based assay from 0 to 120 min. Bars represent mean ± SE for sixteen biological replicates (**A-E**) Data were analysed using single-factor ANOVA and Tukey’s post hoc test (P<0.05). Columns sharing same letter are not statistically different.

Within the group of 593 commonly upregulated genes between two microarray experiments, 52 genes encoded RLKs (including 11 LRR-RLKs, 7 LeCRKs and 1 LysM-RK) and 2 encoded RLPs (S4 and S5 Data). Out of 52 candidate RLKs, we selected homozygous loss-of-function T-DNA mutants for ten genes (from five different RLK families), including those coding for three LRR-RLKs and one LeCRK. Confirmed loss-of-function mutants were then screened for infection against *H*. *schachtii*. Of particular interest, we found one LRR-RLK mutant, termed NILR1 (NEMATODE-INDUCED LRR-RLK 1; NILR1, At1g74360), which showed a consistent increase in the number of female nematodes as compared with Col-0 (Fig 5A and Fig F and G in S1 Text). In comparison to *nilr1-1*, the loss-of-function mutant for *NILR2* (AT1G53430) did not show any change in susceptibility to nematodes (Fig 5A). Based on our data with Proteinase K and BAK1, we hypothesized that NILR1 may be a PRR involved in the perception of nematodes. Therefore, this study focused on the characterization of *NILR1 and NILR2*, while other candidate genes will be described elsewhere.

To test NILR1’s involvement in nematode perception other than *H*. *schachtii*, we analysed *nilr1-1* mutants for infection with root-knot nematode *M*. *incognita*. Our data showed that *nilr1-1* was significantly more susceptible to *M*. *incognita* than Col-0. In comparison, there was no change in susceptibility of *nilr2-1* to *M*. *incognita* (Fig 5B). To investigate whether enhanced susceptibility of *nilr1-1* to nematodes is due to impairment in PTI responses, we performed ROS burst assays on root segments from Col-0 and *nilr1-1* upon treatment with NemaWater from two different nematode species (*H*. *schachtii* and *M*. *incognita*). Notably, the NemaWater-induced ROS burst was strongly reduced in *nilr1-1* (Fig 5C and Fig H in S1 Text). Similar results were obtained in seedling growth inhibition assays (Fig 5D and Fig I in S1 Text). We also tested *nilr2-1* for seedling growth inhibition and ROS burst induction upon treatment with NemaWater. We found that even though ROS production was reduced in *nilr2-1* upon *Hs*NemaWater treatment, the growth of these plants was inhibited to the same extent as Col-0 (Fig 5E and 5F and Fig I in S1 Text). Next, we isolated an additional homozygous knock-out T-DNA line for NILR1 (*nilr1-2*) and analysed it for infection by *H*. *schachtii* and production of ROS burst upon *Hs*NemaWater treatment (Fig J-L in S1 Text). We observed that *nilr1-2* plants were impaired in ROS production and were also significantly more susceptible to *H*. *schachtii* as compared to Col-0 (Fig K-L in S1 Text). Together our results show that NILR1 is an important component of host immune responses that are activated upon nematode infection.

### NILR1 is widely conserved in dicotyledonous plants

**Fig 6:**
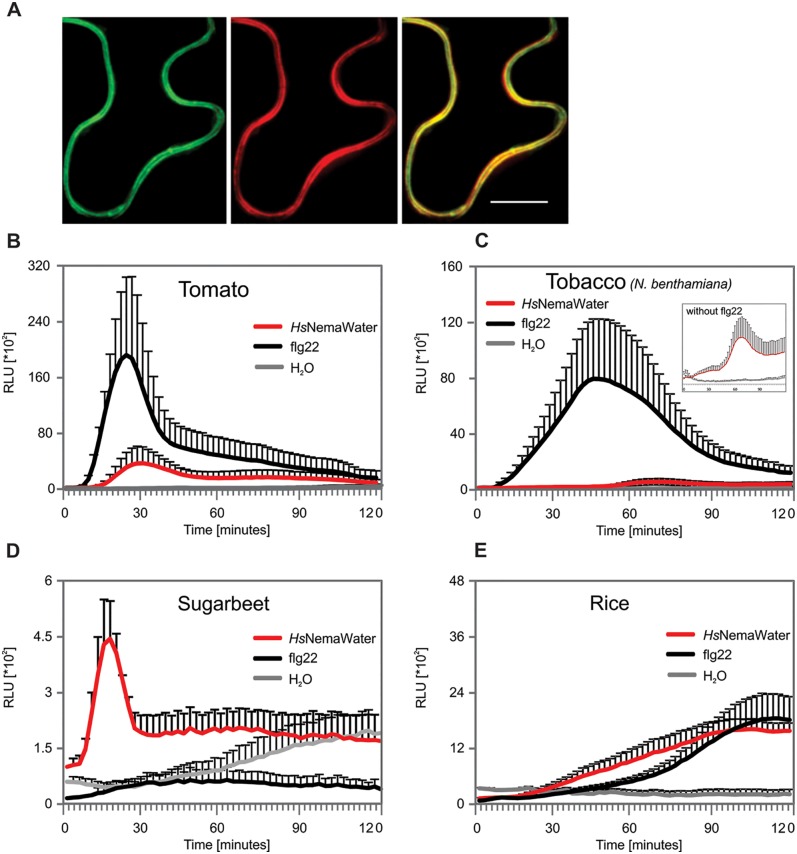
NILR1 is localised in plasma membrane. (A) Confocal microscopy of epidermis of *Nicotianna benthamiana* transiently expressing *35S*:*NILR1-GFP* and plasma membrane marker *35S*:*PIP2A-mCherry*. Scale, 50 μm. (B-E) Leaf discs from tomato (B), *N*. *benthamiana* (C), sugarbeet (D) and rice plants were treated with water, *Hs*NemaWater or flg22 and ROS burst was measured using L-012 based assay from 0 to 120 min. Bars represent mean ± SE for three technical replicates. Experiment was repeated three times with same results. RLU, relative light units.

NILR1 is closely related to LRR-RLK BRI1, belonging to the subfamily X of LRR-RLKs [32]. NILR1 encodes a serine/threonine kinase with 1,106 amino acid residues (predicted molecular weight 121.8 kDa) and shows all of the characteristics of an LRR-RLK. NILR1 has been suggested to have an extracellular domain with 22 tandem copies of LRRs, which are interrupted by a 76-amino acid island located between LRR17 and LRR18. The island domain of NILR1 is longer than those of BRI1 and contains a cysteine cluster with the pattern of C*x*_25_C*x*_16_C, which is followed by a transmembrane domain and a cytoplasmic kinase domain (Fig M-N in S1 Text) [31]. Moreover, a pair of cysteines at the amino terminal flanks NILR1’s LRR domain with the characteristic spacing formerly observed in several plant LRR-RLKs [33]. Previous analysis has shown that NILR1 is presumably localised to the cell membrane, and that homologs are conserved among ten different species of flowering plants [32]. To gain further insights into molecular functions of NILR1, we determined its subcellular localization by confocal microscopy transiently expressing 35S::*NILR1-GFP* in the epidermis of *Nicotianna benthamiana*. We detected a strong GFP signal at the plasma membrane (PM) (Fig 6A). The PM localization of NILR1 was confirmed by co-localization with PM marker (see Methods for details). To investigate the conservation of NILR1, we conducted a BLAST search using ECD’s amino acid sequence of NILR1 against non-redundant protein sequences of all land plants. We detected homologues of NILR1 among different species of the *Brassicaceae* family. Additionally, orthologues of NILR1 were found to be widely conserved in the genome of various dicotyledonous as well as monocotyledonous plant species. (Fig O in S1 Text). To further determine whether NILR1 is conserved across the plant kingdom and to test for effects of NemaWater, we measured the ROS burst upon *Hs*NemaWater treatment in the dicotyledonous tomato, sugar beet (*Beta vulgaris*) and tobacco (*Nicotianna benthamiana*), as well as in monocotyledonous rice (*Oryza sativa*). We detected a strong ROS burst in sugar beet and tomato (Fig 6B and 6C), the magnitude of ROS burst was delayed and reduced in *N*. *benthamiana* (Fig 6D). In comparison to dicotyledonous, experiments with monocotyledonous rice showed that NemaWater induce a ROS burst, which was above the water control (Fig 6E). However, this burst was strongly delayed and was not consistent across several experiments.

A further exploration of publicly available Arabidopsis expression data through the eFP browser [33] revealed that *NILR1* is only moderately expressed in sepals and in senescent leaves under controlled growth conditions. However, *NILR1* expression is upregulated in response to biotic stresses such as *Botrytis cinerea*, *Phytophthora infestans* and non-adapted *Pseudomonas syringae* strains (Fig P and Q in S1 Text). Also NILR1 shows a low basal expression in various root tissues but displays a relatively high expression in endodermis, pericycle and stele [34]. The overall structure of NILR1 and its similarity to BRI1 supports its role as a surface-localised receptor that is involved in the perception of extracellular signals.

## Discussion

In comparison to other pathosystems, not much is known about the importance of PTI in host defense against nematodes. In fact, no PRR involved in nematode perception has thus far been characterized. Additionally, so far only ascarosides have been recently shown to act as NAMPs. On the other hand, a number of nematode resistance genes (*R-genes*) either at the cell surface or inside cells have been characterised [22, 23]. In the present study, we provide insights into the molecular events associated with the basal resistance of plants to nematodes. We demonstrate that PTI-like responses are activated upon nematode infection and that they contribute significantly to basal resistance against nematodes.

The observation that cyst nematode infection induces the activation of a number of JA biosynthesis and signalling genes during migratory stages is supported with biochemical measurements showing an elevated amount of JA in Arabidopsis roots 24 hours after nematode infection [24]. In contrast to JA there was no strong activation of SA signalling in our transcriptome data during migratory stages. Nevertheless, a slight increase in some SA biosynthesis and signalling genes was observed. Intriguingly, plants that are deficient in different aspects of SA-signalling and biosynthesis have been shown to be more susceptible to cyst nematode infection [35]. These observations raise the question as to whether JA activation in roots upon nematode infection is only because of wounding during migration. Remarkably, we observed the same pattern of JA activation in roots upon treatment with *Hs*NemaWater indicating that JA activation is an important component of defense responses that are activated upon nematode recognition and is not only correlated to wounding. This hypothesis contradicts the general view that SA plays a more prominent role against biotrophs while JA/ET appears to be more important in resistance against necrotrophic pathogens and herbivorous insects [36–38]. This view, however, is mainly based on observations with leaf pathogens, whereas only limited information is available on the role of plant hormones in defense against root pathogens [39]. It may be that JA plays a more dominant role in the plant-pathogen interactions in roots. This hypothesis is supported by experiments on rice plants that indicated a key role for JA during interaction with root-knot nematodes [40]. Unlike the migratory phase, a number of studies addressing changes in gene expression during the sedentary phase of cyst and root-knot nematodes infection revealed a strong suppression of host defence responses [4–6]. Based on data from the current study and previous literature, we concluded that nematode invasion activates PTI responses, which are suppressed during later stages of nutrient acquisition and feeding site development. Indeed, an increasing number of nematode effectors involved in suppression of PTI have been characterised during last few years [8, 10, 18, 22, 23].

We observed that NemaWater treatment triggers responses, including ROS burst, immune gene expression and seedling growth inhibition that are characteristic of PTI. In addition, plants treated with NemaWater were more resistant to nematodes compared with water-treated control plants. On the basis of these data we propose that NemaWater contains elicitor/s that is/are perceived by plant surface-localised receptors leading to activation of PTI.

The fact that NemaWater derived from two different nematode species induces similar responses suggests that the elicitor component/s is/are conserved among different nematode species. Although the identity of the elicitor in NemaWater remains unknown, it is likely to be a heat-sensitive protein since treatment with heat as well as with Proteinase K strongly reduced its activity. Nevertheless, the residual growth inhibition in spite of addition of Proteinase K in NemaWater hints towards the possibility of an additional non-proteinaceous NAMP in NemaWater. However, it is also plausible that the residual growth inhibition is caused by Proteinase K itself. This view is supported by our data (Fig 4A) and some previous studies where a slight ROS burst was observed upon Proteinase K treatment alone [27].

NemaWater-induced responses are dependent on BAK1, which has been shown to act as a co-receptor for LRR-type PRRs, which typically detect proteinaceous ligands [12, 15, 17]. Even though we hypothesise that the NemaWater-derived elicitor/s is/are perceived by a surface-localized receptor, the possibility remains that such elicitor/s may not come into contact with host plants during infection. However, the fact that NemaWater was produced by incubating the nematodes without any further treatment strongly supports the idea that the elicitor is naturally secreted into the environment. It is also possible that the treatment of seedlings with NemaWater leads to the release of plant endogenous elicitors (DAMPs), which are again sensed by plants leading to the activation of PTI responses. However, since diluting NemaWater reduced only the magnitude but did not slow down the kinetics of ROS burst and thus makes it unlikely that a NemaWater induced DAMP is responsible for activation of PTI responses. Regardless of the origin of elicitor, it is clear that induction of PTI responses involves a component of NemaWater (therefore a NAMP) and is not only due to direct mechanical wounding by nematodes.

Loss of *NILR1* expression enhances the susceptibility of plants to nematodes suggesting that it is involved in the recognition of nematode-associated patterns. We propose that NILR1 is a PRR (or a component of a PRR complex) that recognises a NAMP leading to the activation of PTI responses. This hypothesis is supported by experiments showing that *nilr1-1* is defective in the ROS burst as well as in seedling growth inhibition upon NemaWater treatment compared with Col-0. Notably, *nilr1-1 and nilr1-2* did not respond differently to flg22 as compared with Col-0. On the other hand, *bak1-5* was defective in PTI activation in response to both flg22 and NemaWater indicating a BAK1-mediated role for NILR1 in nematode recognition. In comparison to *nilr1 (nilr1-1*, *nilr1-2)*, *nilr2-1* did not show any change in susceptibility to neither cyst nor to root-knot nematodes compared to Col-0. Similarly, there was no change in seedling growth inhibition as compared with Col-0. Nevertheless, activation of ROS burst upon NemaWater treatment was decreased in *nilr2-1* as compared with Col-0. This seemingly contradictory observation raises the question as to whether NILR2 also plays a role in perception of nematodes. A possible explanation could be that knocking out NILR2 may alter receptor complex formation and function, which selectively influence downstream signalling pathways without substantially influencing plant susceptibility to nematodes. This hypothesis also predicts that distinct signalling pathways that are activated during nematode perception may lead to diverse signalling outputs independently from each other. In fact, a recent study suggests activation of BAK1-dependent and BAK1-independent PTI pathways in response to RKN infection [19].

In conclusion, the identification of NILR1 as an LRR-RLK required for NemaWater-induced immune responses and basal resistance to nematodes is a major step forward in understanding of the molecular mechanisms underlying plant-nematode interactions. Moreover, the wide distribution of NILR1 among monocot and dicot plants is different from the majority of currently known PRRs and provides a unique opportunity for manipulation. However, sequence similarity does not necessarily indicate similar functions. It is therefore plausible that some of these homologues represent BRI1 or similar receptors and appeared in our analysis due to close similarity between NILR1 and BRI1. In fact, absence of a consistent ROS burst in rice plants upon NemaWater treatment hints that rice plants may not encode a functional NILR1. However, it is also possible that production of ROS burst upon treatment with NemaWater in some plant species such as rice requires further optimisation. A more detailed study would be needed to investigate this aspect.

Future work will focus on the purification and identification of elicitor/s present in NemaWater that are recognised in an NILR1-dependent manner. Further, conservation and function of NILR1 in various crop plants will be investigated. This will not only help in increasing our understanding of induced immune responses, but also provide potential opportunities to breed or engineer durable resistance against nematodes.

## Materials and methods

### Plant growth and nematode infection

*Arabidopsis thaliana* seeds were sterilized with 0.6% sodium hypochlorite and grown in Petri dishes containing agar medium supplemented with modified Knop’s nutrient medium under the previously described conditions [41, 42]. The infection assays with cyst nematodes were performed as previously described [41]. Briefly, 60–70 J2s of *H*. *schachtii* were inoculated to the surface of an agar Knop medium containing 12-days-old plants under sterile conditions. For each experiment, 15–20 plants were used per genotype and experiments were repeated at least three times independently. The number of females per plant was counted at 14 days after inoculation (dai). For each experiment, 15–20 plants were used per genotype, and experiments were repeated at least three times independently.

For infection assays with root-knot nematodes, approximately 100 J2s of *M*. *incognita* were inoculated to the surface of agar MS-Gelrite medium containing 12-day-old plants and number of galls was counted at 21 dpi. *M*. *incognita* was propagated on greenhouse cultures of tomato (*Solanum lycopersicum cv*. *Moneymaker*) plants. Galls on roots of tomato were cut into smaller pieces of approximately 1 cm, crushed, and incubated for 3 min in 1.5% NaOCl_2_. Subsequently, the suspension was passed through a series of sieves to separate nematode eggs from root pieces. Eggs were collected in a 25 μm sieve. For surface sterilisation, eggs were incubated in a 10% NaOCl_2_ for 3 minutes and washed with abundant sterile water. The clean egg suspension was further washed with 150 μL Nystatin (10,000 U/ mL) and 2mL gentamycin sulphate (22.5 mg/mL) in a total volume of 30 mL. The suspension was stored at RT in darkness. Freshly hatched J2s were rinsed in water, incubated for 20 minutes in 0.5% (w/v) streptomycin-penicillin and 0.1% (w/v) ampicillin-gentamycin solution and for 3 minutes in 0.1% (v/v) chlorhexidine and washed three times with liberal amounts of sterile autoclaved water. For each experiment, 15–20 plants were used per genotype, and experiments were repeated at least three times independently.

### Gene expression analysis at the nematode migratory stage

Ten hours after inoculation with *H*. *schachtii*, small root segments containing nematodes with moving stylets were marked under the binocular. Movement of stylet indicates the migration phase of nematodes. The infected area around nematode head was then dissected. Corresponding root segments from uninfected plants were used as a control. RNA was extracted using a Nucleospin RNA extraction kit (Macherey-Nagel, Durren, Germany) according to the manufacturer’s instructions. The quality and quantity of RNA was analysed using an Agilent Bioanalyzer (Agilent Technologies, Santa Clara, CA, USA) and a Nanodrop (Thermo Fisher Scientific, Waltham, MA, USA) respectively. The cDNA synthesis was performed with NuGEN’s Applause 3’Amp System (NuGEN, San Carlos, CA, USA) according to the manufacturers’ instructions. NuGEN’s Encore Biotin Module (NuGEN) was used to fragment cDNA. Hybridization, washing and scanning were performed according to the Affymetrix 30 GeneChip Expression Analysis Technical Manual (Affymetrix, Santa Clara, CA, USA). Three chips each were hybridized with control and infected samples, with each microarray representing an independent biological replicate. The primary data analysis was performed with the Affymetrix Expression Console v1 software using the MAS5 algorithm.

### NemaWater production and gene expression analysis upon NemaWater treatment

Approximately 300 brown cysts were collected from nematode stock culture, which was maintained on mustard roots under sterile conditions. These cysts were incubated in 3 mM ZnCl_2_ in funnels (hatching chambers) to induce hatching. Before collection of J2s, the hatching chamber was checked for microbial contamination. After seven days, J2s were collected in a falcon tube containing double distilled autoclave water. The mixture of nematode in ZnCl_2_ was spinned at 800 rpm for 3 min and supernatant was discarded. Afterwards, 1 ml of 0.05% HgCl_2_ was added and nematodes were incubated in it for 3 min to surface-sterilize them. HgCl_2_ was then removed and autoclaved double distilled water was added in excess (approximately 30 ml). The J2s were left in water for three min to wash them and remove HgCl_2_. After 3 min, nematodes were spinned down at 800 rpm for 3min and the entire washing step was repeated three times.

Approximately 40,000 sterile J2s *of H*. *schachtii* were incubated in 2 ml dd H_2_O for 24 hours at room temperature with continuous shaking. Afterwards, the nematode-water mixture was briefly centrifuged at 800 rpm for 2 minutes. The supernatant was removed to a new Eppendorf tube and was labelled as NemaWater. All steps of NemaWater production were performed under sterile conditions. Twelve-days-old Arabidopsis plants grown in Knop medium, as described above, were removed from agar plates and incubated in NemaWater for one hour each. Whole roots from 10 plants were cut and frozen in liquid nitrogen. Arabidopsis roots treated only with dd H_2_O were used as a control. Three biological replicates were performed. RNA was extracted, amplified and hybridised to perform a microarray analysis, as described above. Three chips for each were hybridised for a control and for NemaWater treated samples, with each microarray representing an independent biological replicate.

### Statistical analysis of microarray data

Affymetrix.CDF and.CEL files were loaded into the Windows GUI program RMAExpress (http://rmaexpress.bmbolstad.com/) for background correction, normalisation (quantile) and summarisation (median polish). After normalisation, the computed robust multichip average (RMA) expression values were exported as a log scale to a text file. Probe set annotations were performed by downloading Affymetrix mapping files matching array element identifiers to AGI loci from ARBC (http://www.arabidopsis.org). All genes that were more than 1.5 fold differentially regulated (t-test; P < 0.05) were pre-selected for further analysis using False discover rate at 5%.

### Validation of microarray chip data upon NemaWater treatment

To validate the microarray expression data, 11 up- and two down-regulated genes were randomly selected. The samples were collected in the same manner as the microarrays analysis for NemaWater. RNA was extracted using a Nucleospin RNA Xs (Macherey- Nagel, Germany) kit according to the manufacturer’s instructions. cDNA was synthesized using a High Capacity cDNA Reverse Transcription Kit (Life technologies cat.no. 4368814), according to the manufacturer’s instructions. The transcript abundance of targeted genes was analysed using the Stepone Plus Real-Time PCR System (Applied Biosystems, USA). Each sample contained 10 μL of Fast SYBR Green qPCR Master Mix with uracil-DNA, glycosylase, and 6-carboxy-x-rhodamine (Invitrogen), 2 mM MgCl_2_, 0.5 μL of forward and 0.5 μL of reverse primers (10 μM), 2 μL of complementary DNA (cDNA) and water in 20 μL of total reaction volume. Samples were analysed in three technical replicates. To serve as an internal control, 18S genes were used. Relative expression was calculated as described previously [43], by which the expression of the target gene was normalized to 18S to calculate fold change. All primer sequences are listed in S6 Data.

### Genotyping and expression analysis of knock-out mutants

Single T-DNA inserted knockout mutants for selected genes (AT1G74360: *nilr1-1*, SAIL_859_H01, *nilr1-2*, GK-179E06; AT1G53430: *nilr2-1*, SALK129312C) were ordered from relevant stock centre. The homozygosity of mutants was confirmed via PCR using primers given in S6 Data. The homozygous mutants were confirmed to be completely absent from expression through RT-PCR with primers given in S6 Data.

### Oxidative burst assay

The production of an ROS burst was evaluated using a modified protocol adapted from previous work [27]. Small root segments (approx. 0.5 cm) were cut from 12-days-old plants and floated in ddH_2_O for 12 hours. Afterwards, the root segments were transferred to a well in a 96-well plate containing 15 μl of 20 μg/ml horseradish peroxidase and 35 μl of 0.01M 8-Amino-5-chloro-2,3-dihydro-7-phenyl-pyrido[3,4-d] pyridazine sodium salt (L-012, Wako Chemicals). Next, 50 μl of either 1 μM flg22 or NemaWater was added to the individual wells. The experiments were performed in four technical replicates, and ddH_2_O was used as a negative control. Light emission was measured as relative light units in a 96-well luminometer (Mithras LB 940; Berthold Technologies) over 120 minutes and analysed using instrument software and Microsoft Office Excel. For experiments with Proteinase K, 100 μl of Proteinase K was added to 1 ml of NemaWater or flg22, and the mixture was incubated at 37°C for 4 hours. For heat treatment, samples were incubated at 90°C for 30 min. ddH_2_O was used as a negative control. The experiments were performed in three technical replicates and independently repeated multiple times as indicated in figure legends.

### Growth inhibition assay

Arabidopsis plants were grown in Knop medium, as described above. Five-days-old plants were transferred to a well in a 6-well plate containing a liquid MS medium supplemented with either 1 ml of 1 μM flg22 or NemaWater. ddH_2_O was used as a negative control. Fresh weight and length of the roots were measured 7 days after they were transferred to MS medium. The experiments were performed in three technical replicates and independently repeated multiple times as indicated in figure legends.

### *In silico* structural analysis and localization of NILR1

The amino acid sequence for ECD of NILR1 was used to blast against all land plants sequences resulting in 318 hits across kingdom. Representative sequences from 44 unique species were used to generate a multiple alignment file. A Gblock function was used to refine alignment, and a maximum-likelihood analysis was performed with the PHYML software [44]. A nonparametric approximate likelihood ratio test was used for branch support as an alternative to usual bootstrapping procedure [45].

ECD sequence of NILR1 was used to search the SWISS-MODEL template library (SMTL version 2016-03-23, PDB release 2016-03-18) with Blast and HHBlits for evolutionary related matching structures matching [46–48]. NILR1 match best with BRASSINOSTEROID INSENSITIVE 1 (BRI1) and the PDB file from SWISS-MODEL was used to view 3-dimensional structures with NCBI Cn3D [49].

Coding region of NILR1 was amplified without stop codon using gateway forward and reverse primers as given in S6 Data. The amplified fragment was cloned into pDONR207 using BP clonase (Invitrogen) according to manufacturer’s instructions. The resultant pENTRY vector (pENTRY/NILR1) was then used to clone NILR1 into the destination vector pMDC83:CGFP [50] using LR clonase (Invitrogen) according to manufacturer’s instructions. The expression vector (35S:NILR1-GFP) was transformed into *Agrobacterium* strain GV3101 and co-infiltrated together with a plasma membrane mCherry marker *35S*:*PIP2A-mCherry* [51] into epidermis of 6-week old *Nicotianna benthamiana* leaves [52]. The GFP and mCherry signal was detected using a confocal microscope (Zeiss CLSM 710).

## Supporting information

S1 Text **(A)** GO categories preferentially upregulated during migratory stages of nematode infection. **(B)** Inhibition of root growth upon NemaWater treatment. 5-day-old Col-0 seedlings were incubated in water, *Hs*NemaWater or flg22 for seven days. Fresh weight of root was measured at 12 days after germination. Data were analyszed using *t-test*. Asterisk represent significant difference to water-treated control root segments (P<0.05). Hs, *Heterodera schachtii*. **(C)** GO categories preferentially upregulated upon NemaWater treatment. **(D)** An illustration of our method for cyst nematode counting. Each petridish is screened at 14 dpi under the binocular microscope and each female nematode is marked (represented by dots) to calculate rate of infection per plant. **(E)** NemaWater treatment growth inhibition was reduced strongly in *bak1-5*. 5-day-old Col-0 and *bak1-5* seedlings were incubated in water, NemaWater, or flg22 for seven days. Fresh weight of the root was measured at 12 days after germination. Data were analyzed using single-factor ANOVA and Dunnet’s post hoc test (P<0.05). Columns sharing same letter are not statistically different. **(F)** Genotyping for NILR1 and NILR2 mutants. Genomic DNA of Col-0 or knockout lines (*nilr1-1*, *nilr2-1*) was PCR amplified using primers given in S6 Data. The presence or absence of intact wild-type allele is shown. **(G)** RT-PCR for presence or absence of gene expression in Col-0 or knockout mutants. RNA from Col-0 or knockout lines (*nilr1-1*, *nilr2-1*) was extracted to synthesize single stranded cDNA. The presence or absence of expression is shown using primers given in S6 Data. **(H)** Knock-out *nilr1* enhances susceptibility to nematodes. Root segments from Col-0, and *nilr1-1* plants were treated with water, flg22 or NemaWater from *M*. *incognita* (*Mi*NemaWater) and ROS burst was measured using L-012 based assay from 0 to 120 min. Bars represent mean ± SE for twelve biological replicates. **(I)** NemaWater-induced growth inhibition was reduced strongly in *nilr1-1*. 5-day-old Col-0, *nilr1-1and nilr2-1* seedlings were incubated in water, NemaWater, or flg22 for seven days. Fresh weight of the root was measured at 12 days after germination. Data were analyzed using single-factor ANOVA and Dunnet’s post hoc test (P<0.05). Columns sharing same letter are not statistically different. **(J)** Expression analysis of for *nil1-2* mutants. RT-PCR for presence or absence of gene expression in Col-0 or knockout mutants. RNA from Col-0 or knockout line (*nilr1*-2) was extracted to synthesize single stranded cDNA. The presence or absence of expression is shown using primers given in S6 Data. **(K)** Knock-out *nilr1-2* enhances susceptibility to nematodes. Average number of female nematodes per plants in Col-0 and *nilr1-2*. Bars represent mean ± SE for six biological replicates. **(L)** Knock-out *nilr1-2* enhances susceptibility to nematodes. Root segments from Col-0 and *nilr1-2* plants were treated with water, flg22 or NemaWater from *M*. *incognita* (*Mi*NemaWater) and ROS burst was measured using L-012 based assay from 0 to 120 min. Bars represent mean ± SE for three technical replicates. Experiment was repeated three times with similar results. **(M)** NILR1 encodes a LRR receptor kinase. Primary structure of the NILR1 divided into signal peptide; N-terminal containing a pair of cysteine residues (underlined); the LRR domain with LRR consensus residues in grey; the island domain containing a cysteine cluster with the pattern of Cx2Cx16C; the transmembrane domain; and the Ser/Thr kinase domain. **(N)** A putative structural model for ECD of NILR1. The model was built using BRI1 as template. Conserved and similar residues between BRI1 and NILR1 are highlighted as red or blue respectively. Grey color represents additional residues. **(O)** Conservation of NILR1 in land plants. A phylogram tree generated from maximum-likelihood trees construction method based on alignment of sequence spanning NILR1's ECD. The number next to each branch (in brown) indicates a measure of support. The number varies between 0 and 1 where 1 represent maximum. **(P)** Expression of NILR1 during development stages of plants. As revealed by eFP browser. **(Q)** Expression of NILR1 under different biotic stress conditions as revealed by eFP browser [34].
(PDF)

S1 Data Arabidopsis genes differentially regulated *(FDR* <0.05; Fold change >1.5).during migratory stages of nematode infection. Root segments from uninfected roots were used as control. Values indictae fold change compared with control.
(XLSX)

S2 Data Expression data for a selection of Jasmonic Acid- (JA), Ethylene- (ET) and Salicylic Acid genes (SA)-related biosynthesis, signaling and marker genes with fold changes obtained from microarrays analysis representing migratory stages of nematode infection. Values indictae fold change compared with control. Values in green are significantly different *(FDR* <0.05; Fold change >1.5).
(XLSX)

S3 Data Arabidopsis genes differentially regulated *(FDR* <0.05; Fold change >1.5) upon *Hs*NemaWater treatment. Root segments from uninfected roots were used as control. Values indictae fold change compared with control.
(XLSX)

S4 Data A set of commonly upregulated genes between two microarrays (S1 and S3 Data). (XLSX)

S5 Data All RLKs and RLPs differentially commonly upregulated between two microarrays (S1 and S3 Data). (XLSX)

S6 Data Primer sequences used in this study. (DOCX)
