# Supplementary material for: Arabidopsis leucine-rich repeat receptor–like kinase NILR1 is required for induction of innate immunity to parasitic nematodes
Source: PLoS Pathog. 2017 Apr 13;13(4):e1006284. doi: 10.1371/journal.ppat.1006284 (PMC5391088; doi:10.1371/journal.ppat.1006284)
Supplement: S1 Text — (A) GO categories preferentially upregulated during migratory stages of nematode infection. (B) Inhibition of root growth upon NemaWater treatment. 5-day-old Col-0 seedlings were incubated in water, HsNemaWater or flg22 for seven days. Fresh weight of root was measured at 12 days after germination. Data were analyszed using t-test. Asterisk represent significant difference to water-treated control root segments (P<0.05). Hs, Heterodera schachtii. (C) GO categories preferentially upregulated upon NemaWater treatment. (D) An illustration of our method for cyst nematode counting. Each petridish is screened at 14 dpi under the binocular microscope and each female nematode is marked (represented by dots) to calculate rate of infection per plant. (E) NemaWater treatment growth inhibition was reduced strongly in bak1-5. 5-day-old Col-0 and bak1-5 seedlings were incubated in water, NemaWater, or flg22 for seven days. Fresh weight of the root was measured at 12 days after germination. Data were analyzed using single-factor ANOVA and Dunnet’s post hoc test (P<0.05). Columns sharing same letter are not statistically different. (F) Genotyping for NILR1 and NILR2 mutants. Genomic DNA of Col-0 or knockout lines (nilr1-1, nilr2-1) was PCR amplified using primers given in S6 Data. The presence or absence of intact wild-type allele is shown. (G) RT-PCR for presence or absence of gene expression in Col-0 or knockout mutants. RNA from Col-0 or knockout lines (nilr1-1, nilr2-1) was extracted to synthesize single stranded cDNA. The presence or absence of expression is shown using primers given in S6 Data. (H) Knock-out nilr1 enhances susceptibility to nematodes. Root segments from Col-0, and nilr1-1 plants were treated with water, flg22 or NemaWater from M. incognita (MiNemaWater) and ROS burst was measured using L-012 based assay from 0 to 120 min. Bars represent mean ± SE for twelve biological replicates. (I) NemaWater-induced growth inhibition was reduced strongly in nilr1-1. 5-day-o [file ppat.1006284.s001.pdf]

Arabidopsis leucine-rich repeat receptor–like kinase NILR1 is required for induction of innate immunity to parasitic nematodes

Badou Mendy 1*, Mary Wanjiku Wang'ombe 1*, Zoran S. Radakovic 1, Julia Holbein 1, Muhammad Ilyas 1, Divykriti Chopra 1, Nick Holton 2, Cyril Zipfel 2, Florian M. W. Grundler 1†, Shahid Siddique 1†

[1]Rheinische Friedrich-Wilhelms-University of Bonn, INRES – Molecular Phytomedicine, Karlrobert-Kreiten-Straße 13, D-53115 Bonn, Germany

[2] The Sainsbury Laboratory, Norwich Research Park, Norwich, NR4 7UH, UK

* Equal contributions

† Corresponding authors: Dr. Shahid Siddique: siddique@uni-bonn.de and Prof. Florian M. W. Grundler: grundler@uni-bonn.de

S1 Text

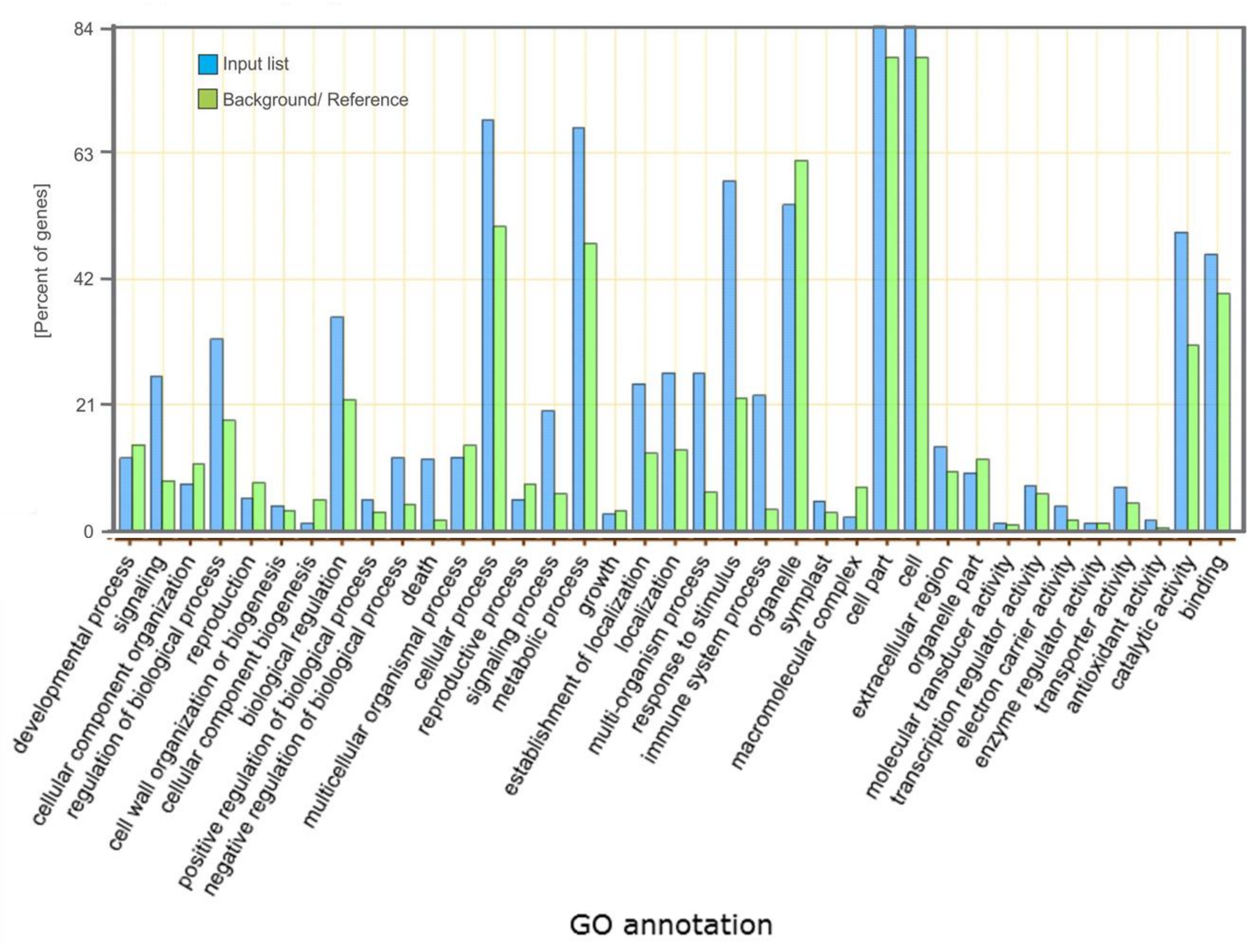

Fig A: GO categories preferentially upregulated during migratory stages of nematode infection.

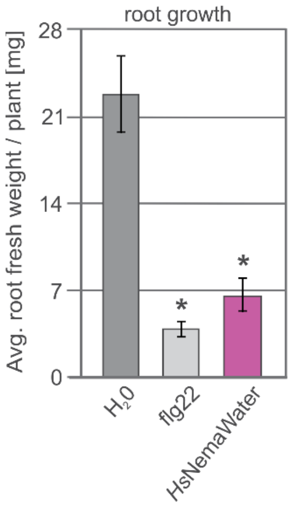

Fig B: Inhibition of root growth upon NemaWater treatment. 5-day-old Col-0 seedlings were incubated in water, flg22, or *Hs*NemaWater for seven days. Fresh weight of root was measured at 12 days after germination. Data were analyzed using *t-test*. Asterisk represent significant difference to water-treated control root segments (P<0.05). Hs, *Heterodera schachtii*.

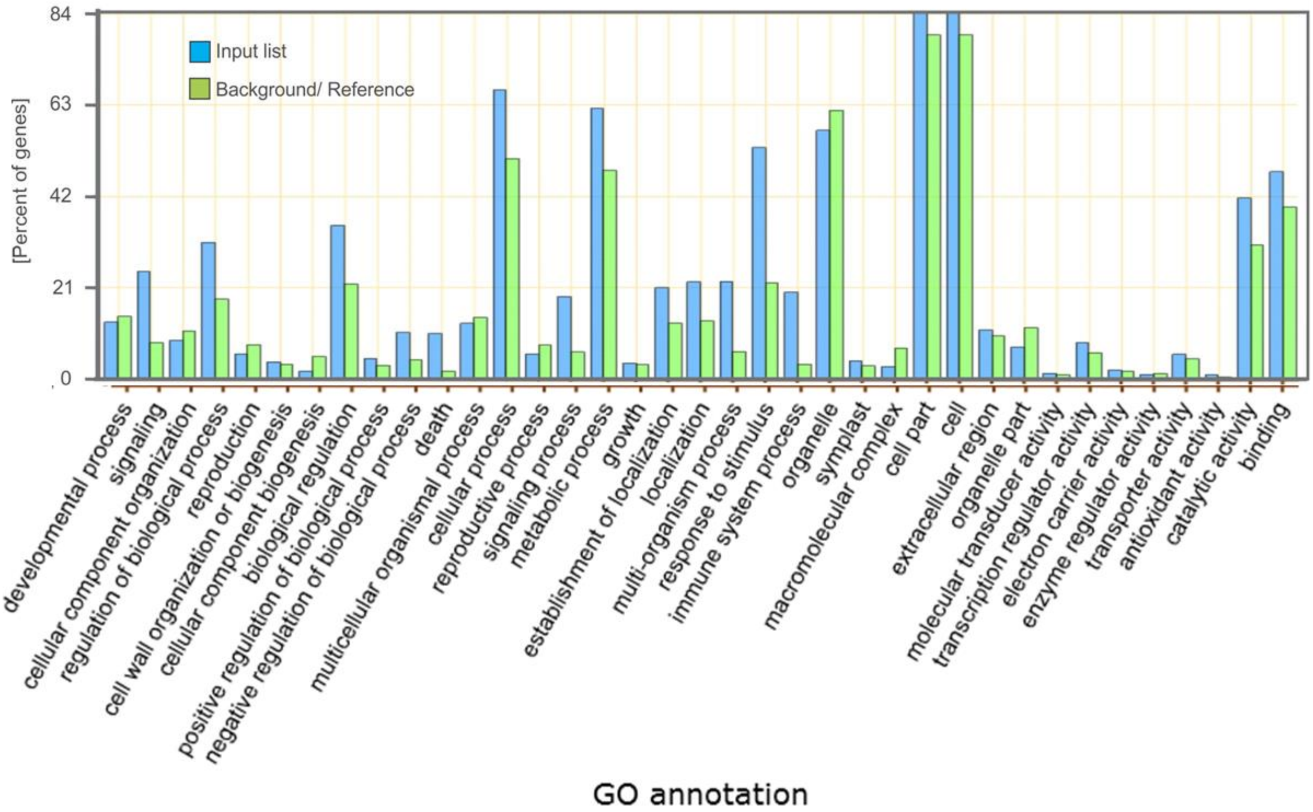

Fig C: GO categories preferentially upregulated upon NemaWater treatment.

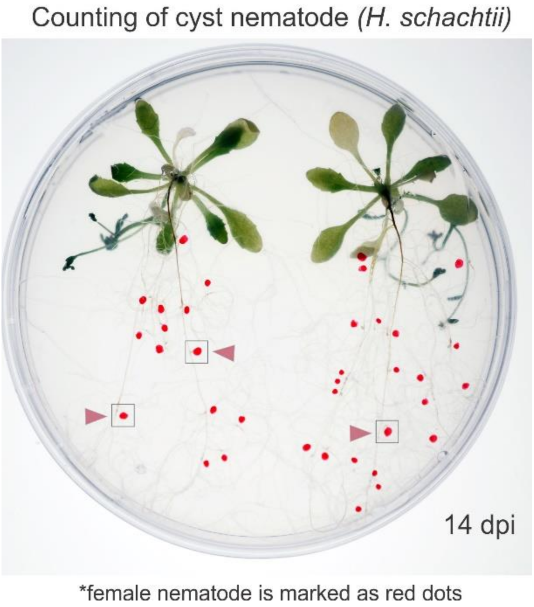

Fig D: An illustration of our method for cyst nematode counting. Each Petri dish is screened at 14 dpi under the binocular microscope and each female nematode is marked (represented by dots) to calculate rate of infection per plant.

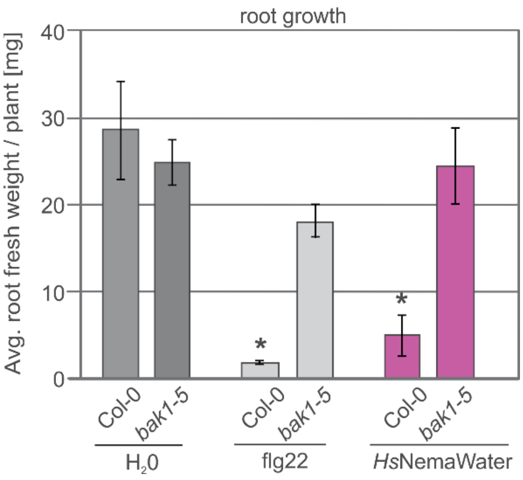

Fig E: Growth inhibition was impaired in *bak1-5* upon NemaWater treatment. 5-day-old Col-0 and *bak1-5* seedlings were incubated in water, flg22, or *Hs*NemaWater for seven days. Fresh weight of the root was measured at 12 days after germination. Data were analyzed using single-factor ANOVA and Dunnet's post hoc test ($P<0.05$).

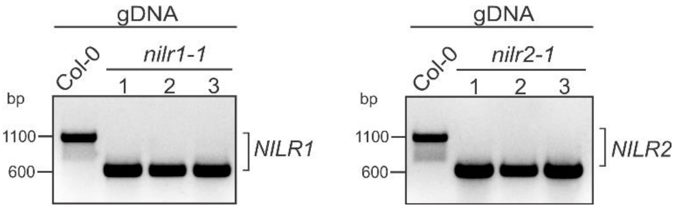

Fig F: Genotyping of NILR1 and NILR2 mutants. Genomic DNA of Col-0 or knockout lines (*nilr1-1*, *nilr2-1*) was PCR amplified using primers given in Dataset 6. The presence or absence of intact wild-type allele is shown.

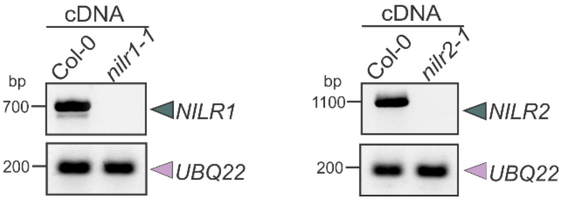

Fig G: RT-PCR for presence or absence of gene expression in Col-0 or knockout mutants. RNA from Col-0 or knockout lines (*nilr1-1*, *nilr2-1*) was extracted to synthesize single stranded cDNA. The presence or absence of expression is shown using primers given in Dataset 6. The upper and lower panel run separately.

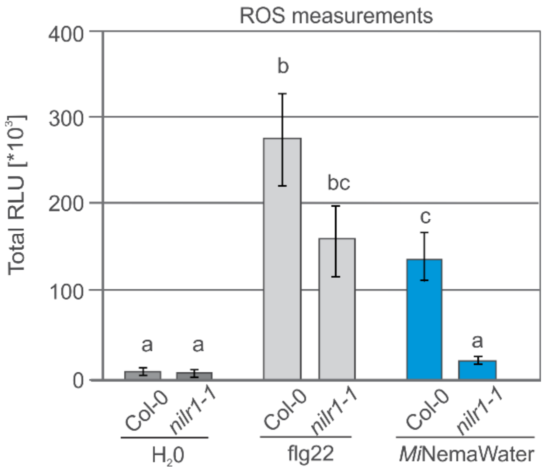

Fig H: Knocking of *NILR1* impair ROS burst to *Mi*NemaWater. Root segments from Col-0, and *nilr1-1* plants were treated with water, flg22 or NemaWater from *M. incognita* (*Mi*NemaWater) and ROS burst was measured using L-012 based assay from 0 to 120 min. Bars represent mean ± SE for twelve biological replicates. Columns sharing same letter are not statistically different.

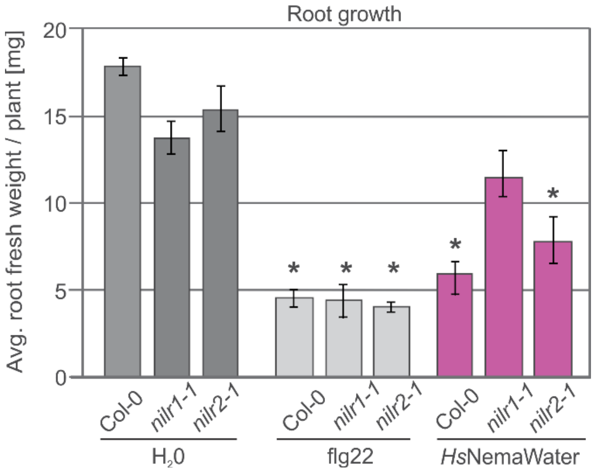

Fig I: NemaWater-induced growth inhibition was reduced strongly in *nilr1-1*. 5-day-old Col-0, *nilr1-1and nilr2-1* seedlings were incubated in water, flg22, or NemaWater for seven days. Fresh weight of the root was measured at 12 days after germination. Data were analyzed using single-factor ANOVA and Dunnet's post hoc test (P<0.05).

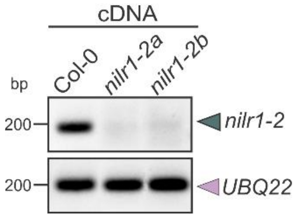

Fig J: Expression analysis of *nilr1-2* mutants. RT-PCR for presence or absence of gene expression in Col-0 or knockout mutants. RNA from Col-0 or knockout line (*nilr1*-2) was extracted to synthesize single stranded cDNA. a and b represent two independent plants. The presence or absence of expression is shown using primers given in Dataset 6.

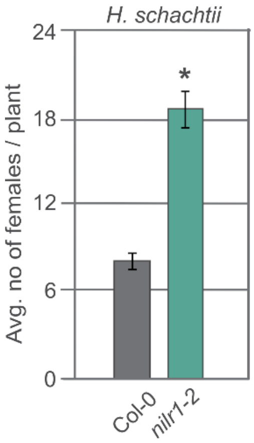

Fig K: Knock-out *nilr1-2* enhances susceptibility to nematodes. Average number of female nematodes per plant in Col-0 and *nilr1-2*. Bars represent mean ± SE for six biological replicates.

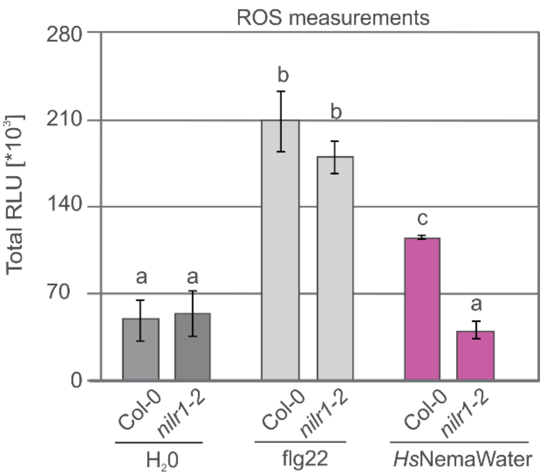

Fig L: Knock-out *nilr1-2* enhances susceptibility to nematodes. Root segments from Col-0, and *nilr1-2* plants were treated with water, flg22 or NemaWater from *H. schachtii* (*Hs*NemaWater) and ROS burst was measured using L-012 based assay from 0 to 120 min. Bars represent mean ± SE for three technical replicates. Experiment was repeated three times with similar results.

Signal peptide 1

TMVTRVIMTDDDSQSLCFLCFLFFFITAIAVAG

N-Terminus

35

DSLDSDREVLLSLKSYLESRNPQNRGLYTEWKMENQDVVCQWP
GIICTPQRSR

LRR domain 1-17

88

VTGINLTDSTISGPLFKNFSALTE
LTYLDLSRNTIEGEIPDDLSRCHN
LKHLNLSHNILEGELSLPGLSN
LEVLDLSLNRITGDIQSSFPLFCNS
LVVANLSTNNFTGRIDDIFNGCRN
LKYVDFSSNRFSGEVWTGFGR
LVEFSVADNHLSGNISASMFRGNCT
LQMLDLSGNAFGGEFPGQVSNCQN
LNVLNLWGNKFTGNIPAEIGSISS
LKGLYLGNNTFSRDIPETLLNLTN
LVFLDLSRNKFGGDIQEIFGRFTQ
VKYLVLHANSYVGGINSSNILKLPN
LSRLDLGYNNFSGQLPTEISQIQS
LKFLILAYNNFSGDIPQEYGNMPG
LQALDLSFNKLTGSIPASFGKLTS
LLWLMLANNSLSGEIPREIGNCTS
LLWFNVANNQLSGRFHPELTRMG

Island domain I

493

SNPSPTFEVNRQNKDKIIAGSGECLAMKRWIPAEFPPFNFVYAILTKKSCRS
LWDHVLKGYGLFPVCSAGSTVRTLKI

LRR domain (18-22)

SAYLQLSGNKFSGEIPASISQMDR
LSTLHLGFNEFEGKLPPEIGQLP
LAFLNLTRNNFSGEIPQEIGNLKC
LQNLDLSFNNFSGNFPTSLNDLNE
LSKFNISYNPFISGAIPTTGQVAT

LXXL[DN]LSXNX[FIL][STE]GX[FIL]PX[SE][FIL][SG]RQNXX

690

FDKDSFLGNPLLRFPSFFNQSGNNTRKISNQVLGNRPRT

Transmembrane domain

729

LLLIWISLALALAFIACLVVSGIVLM

Ser/Thr kinase

755

VVKASREAEIDLLDGSKTRHDMTSSSGGSSPWLSGKIKVIRLDKSTFTYADILK
ATSNFSEERVVGRGGYGTVYRGVLPDGREVAVKKLQREGTEAEKEFRAEMEVLS
ANAFGDWAHPNLVRLYGWCLDGSEKILVHEYMGGGSLEELITDKTKLQWKKRID
IATDVARGLVFLHHECYPSIVHRDVKASNVLLDKHGNARVTDFGLARLLNVGDS
HVSTVIAGTIGYVAPEYGQTWQATTRGDVYSYGVLTMELATGRRAVDGGEECLV
EWARRVMTGNMTAKGSPITLSGTKPGNGAEQMTELLKIGVKCTADHPQARPNMK
EVLAMLVKISGKAELFNGLSSQGYIEM

Fig M: NILR1 encodes a LRR receptor kinase. Primary structure of the NILR1 divided into signal peptide; N-terminal containing a pair of cysteine residues (underlined); the LRR domain with LRR consensus residues in grey; the island domain containing a cysteine cluster with the pattern of Cx2Cx16C; the transmembrane domain; and the Ser/Thr kinase domain.

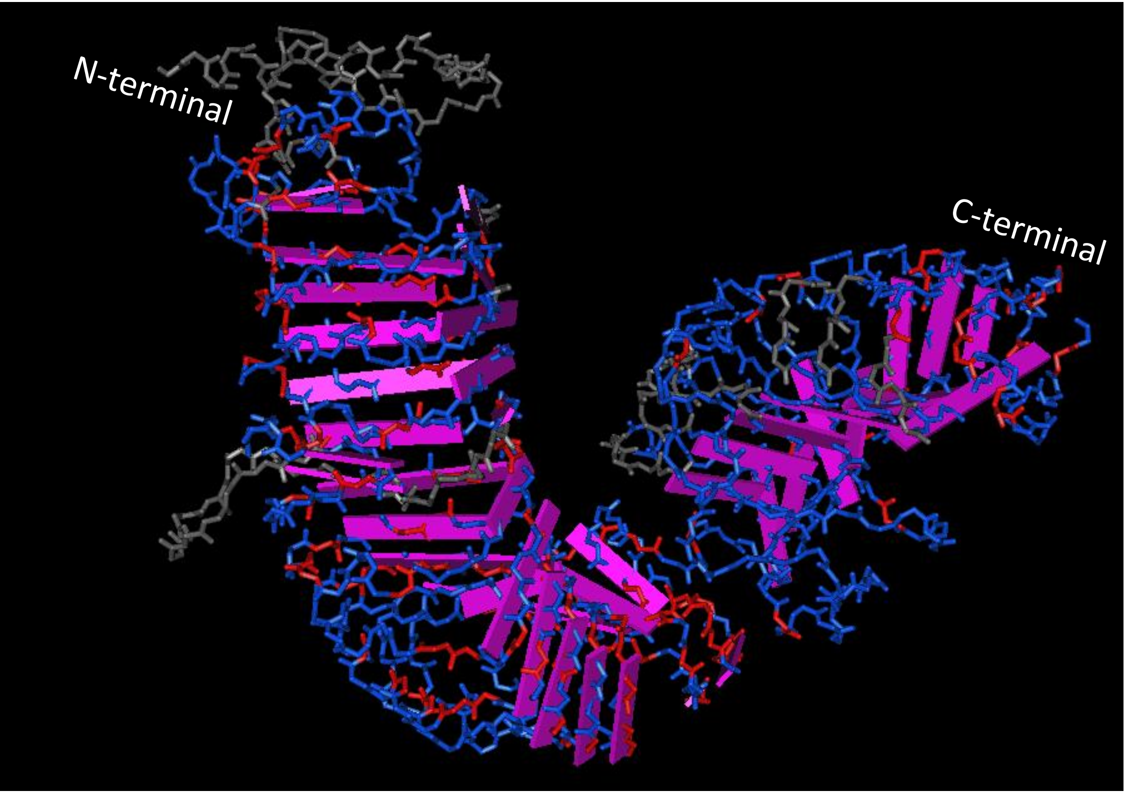

Fig N: A putative structural model for ECD of NILR1**.** The model was built using BRI1 as template. Conserved and similar residues between BRI1 and NILR1 are highlighted as red or blue respectively. Grey color represents additional residues. White dashed box represent Island domain.

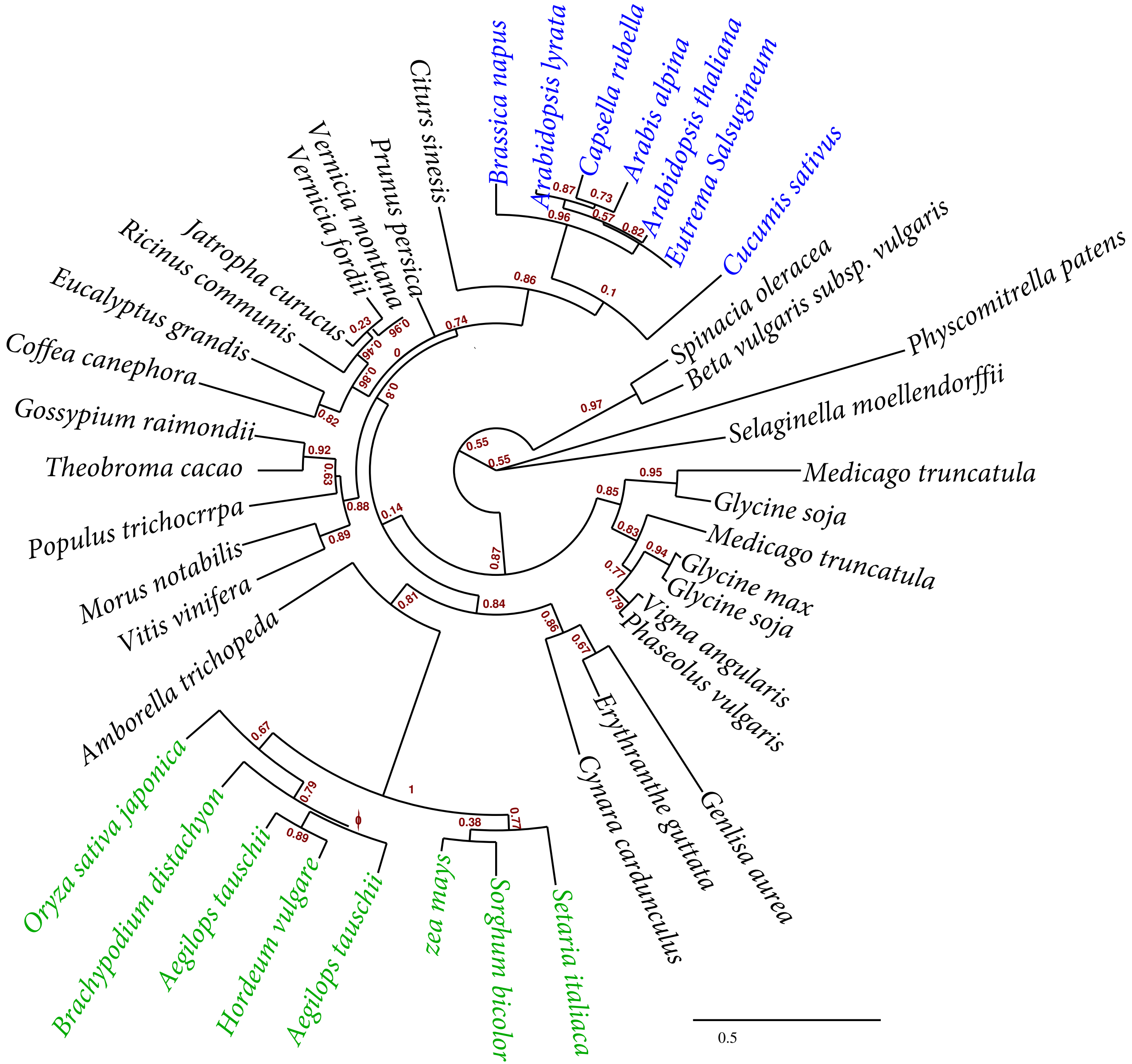

Fig O: Conservation of NILR1 in land plants. A phylogram tree generated from maximum-likelihood trees construction method based on alignment of sequence spanning NILR1's ECD. The number next to each branch (in brown) indicate a measure of support. The number varies between 0 and 1 where 1 represent maximum.

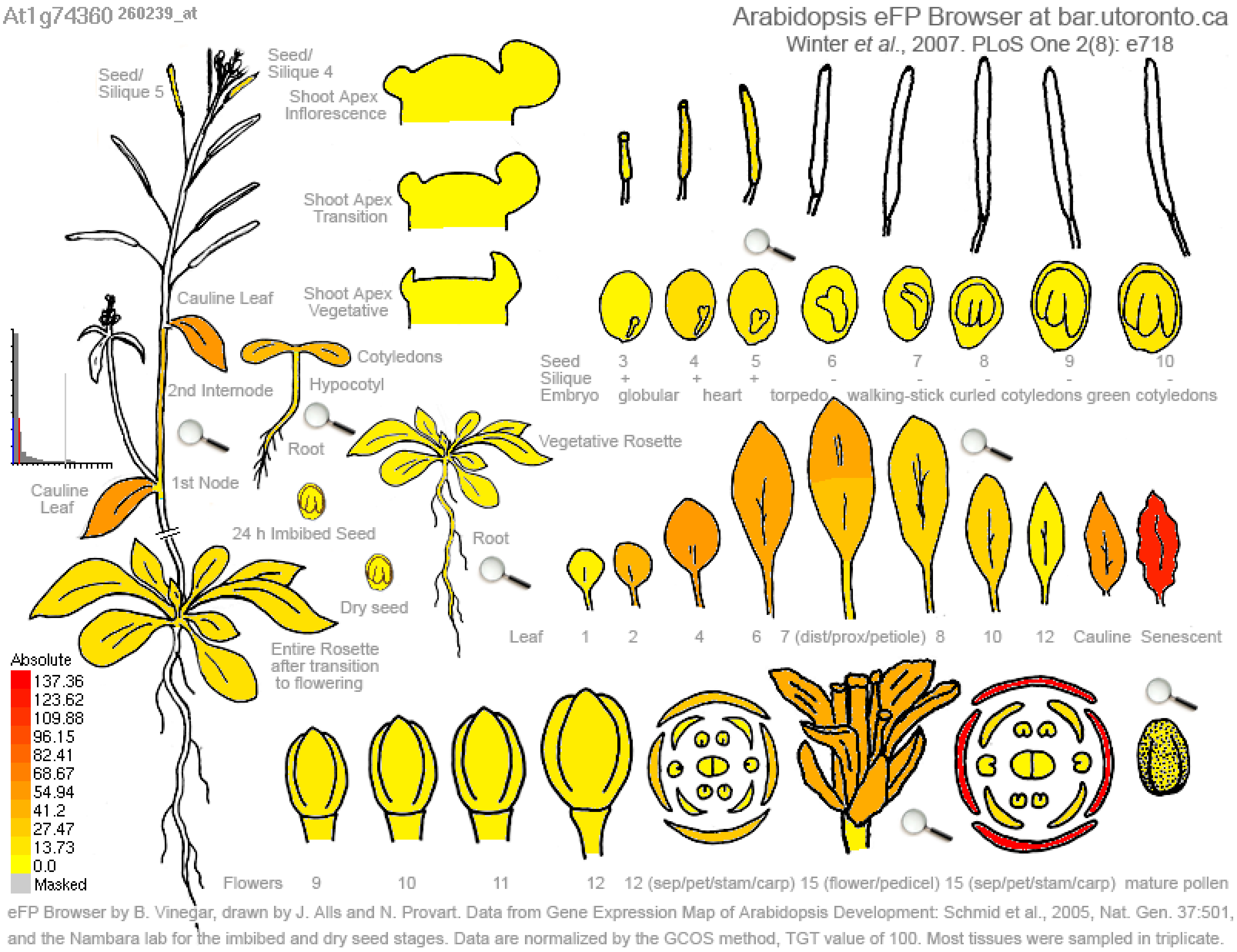

Fig P: Expression of NILR1 during development stages of plants.

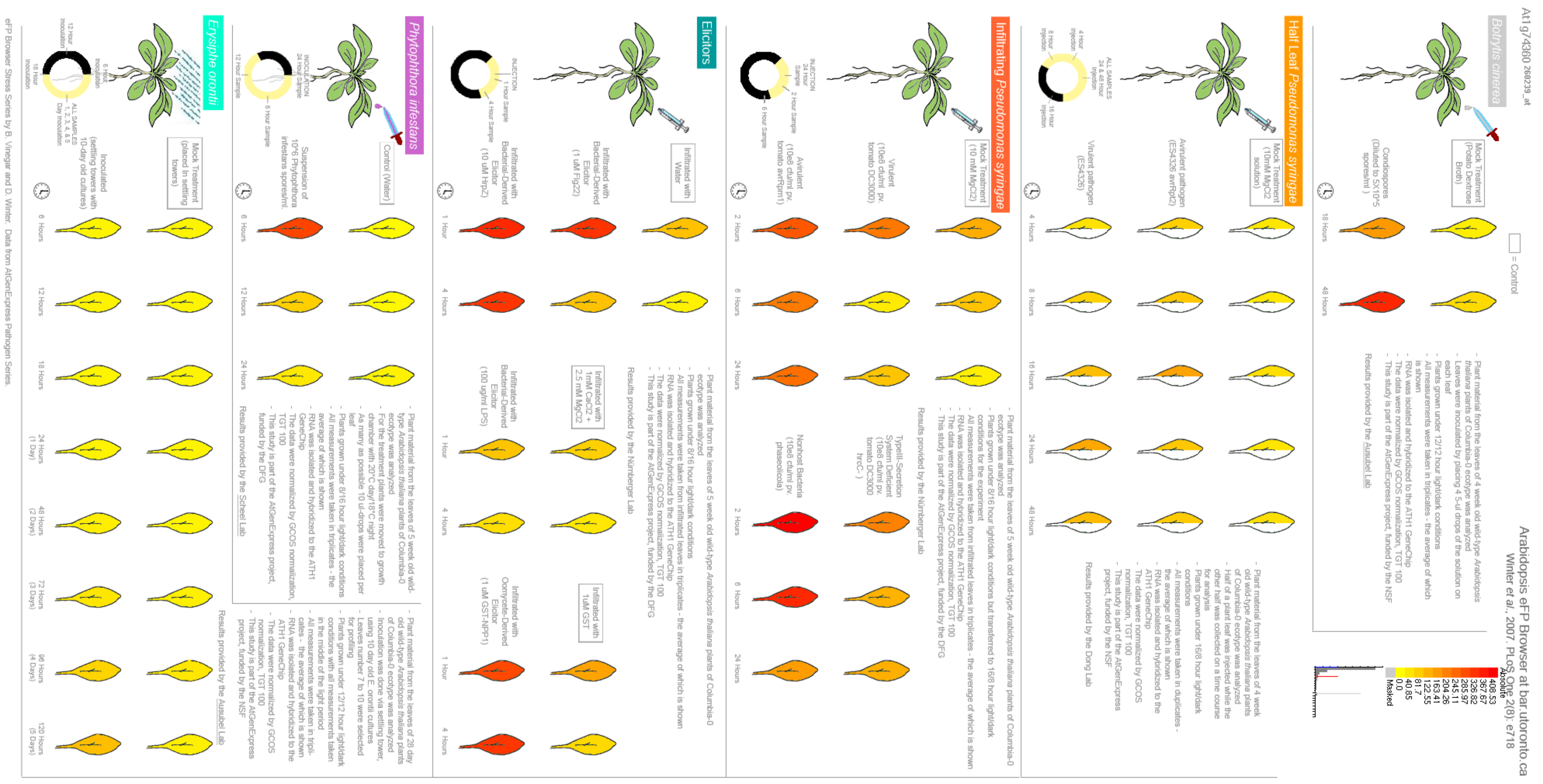

Fig Q: Expression of NILR1 under different biotic stress conditions.
