# Supplementary material for: Arabidopsis leucine-rich repeat receptor–like kinase NILR1 is required for induction of innate immunity to parasitic nematodes
Source: PLoS Pathog. 2017 Apr 13;13(4):e1006284. doi: 10.1371/journal.ppat.1006284 (PMC5391088; doi:10.1371/journal.ppat.1006284)
Supplement: S6 Data — (DOCX) [file ppat.1006284.s007.docx]

| **Locus** | **LEFT PRIMER** | **RIGHT PRIMER** | **Product Size** |
| --- | --- | --- | --- |
| At3g55950-qPCR | AGGAGGCAGAGAAGTGGAAC | CGCTACTTCTCTCCCGTCAT | 171 |
| At4g21390-qPCR | TCATGGGAGATCAGAGGAGC | GCAGCCGAATCTTGAACACA | 111 |
| At1g66880-qPCR | TTACACAGCCACAACCTTGC | GACACTGAGACAAGGCCTCT | 141 |
| At1g69930-qPCR | CCTAGCCCTTTTGTGCTGAG | TTTGTTGCCATGAATGAGGA | 153 |
| At3g46230-qPCR | CGGTCAAGTGGGAAGTTCAT | GACCTCCGGCTTACTCTCCT | 132 |
| At2g38470-qPCR | TGGAGAGAGCATCACACGAC | GTGCTCTGTTTGTGGCGTAA | 108 |
| At5g25930-qPCR | CGGAGACTTATCGGAGCTTG | GGGTGAGATTTCTCCGATCA | 145 |
| At4g23190-qPCR | GGGAAGGATTAATGGGTCGT | TAGTCAATTGCGCTTTGTCG | 193 |
| At1g74360-qPCR | GCCATGGCTATCAGGAAAAA | GCCTCCCCTACCTACCACTC | 118 |
| At5g48540-qPCR | TGCCACAACAAGAAAGGTTG | CGGAGAGAGATGAACGGAAG | 138 |
| At1g11050-qPCR | CGTCTCCCAAATCTCGCTAC | GTTTGGATTCCAGCACAGGT | 149 |
| At1g61590-qPCR | CTCAGCCTGTTGCTGTCAAG | TTCGGGTGTTTTAGCTGTCC | 100 |
| At4g26790-qPCR | CATCCTGAAGCATTCGGTTT | TTGCATCCGAACATGTGAAT | 112 |
| NILR1/Genotyping | AACTTCTCCGCTTTAACGGAG | TGTGCAGTTGCCAATCTCTC | 1114 |
| NILR2/Genotyping | GGGAGTTGCAATTATGCTAGAAG | ATTGGGCATATGTCTTGCAAG | 1018 |
| NILR1, RT-PCR | GAGGCTGGTCGAGTTTTCAG | TGTGCAGTTGCCAATCTCTC | 727 |
| NILR2, RT-PCR | GTCGAGTCAGCCTCCAACTC | AGACCCTCTCTCAAGCACCA | 974 |
| NILR1-2, RT-PCR | AGGTAGGGGAGGCTATGGAA | GTTCGGATGTGCCCAATCTC | 163 |
